# The transcription factor reservoir and chromatin landscape in activated plasmacytoid dendritic cells

**DOI:** 10.1186/s12863-021-00991-2

**Published:** 2021-09-20

**Authors:** Ritu Mann-Nüttel, Shafaqat Ali, Patrick Petzsch, Karl Köhrer, Judith Alferink, Stefanie Scheu

**Affiliations:** 1 Institute of Medical Microbiology and Hospital Hygiene, University of Düsseldorf, Düsseldorf, Germany; 2 Cells in Motion Interfaculty Centre, Münster, Germany; 3 Department of Mental Health, University of Münster, Münster, Germany; 4 Biological and Medical Research Center (BMFZ), Medical Faculty, University of Düsseldorf, Düsseldorf, Germany

**Keywords:** Transcription factors, Plasmacytoid dendritic cells, TLR9, Gene expression analysis, Next generation sequencing, ATAC-Seq

## Abstract

**Background:**

Transcription factors (TFs) control gene expression by direct binding to regulatory regions of target genes but also by impacting chromatin landscapes and modulating DNA accessibility for other TFs. In recent years several TFs have been defined that control cell fate decisions and effector functions in the immune system. Plasmacytoid dendritic cells (pDCs) are an immune cell type with the unique capacity to produce high amounts of type I interferons quickly in response to contact with viral components. Hereby, this cell type is involved in anti-infectious immune responses but also in the development of inflammatory and autoimmune diseases. To date, the global TF reservoir in pDCs early after activation remains to be fully characterized.

**Results:**

To fill this gap, we have performed a comprehensive analysis in naïve versus TLR9-activated murine pDCs in a time course study covering early timepoints after stimulation (2 h, 6 h, 12 h) integrating gene expression (RNA-Seq) and chromatin landscape (ATAC-Seq) studies. To unravel the biological processes underlying the changes in TF expression on a global scale gene ontology (GO) analyses were performed. We found that 70% of all genes annotated as TFs in the mouse genome (1014 out of 1636) are expressed in pDCs for at least one stimulation time point and are covering a wide range of TF classes defined by their specific DNA binding mechanisms. GO analysis revealed involvement of TLR9-induced TFs in epigenetic modulation, NFκB and JAK-STAT signaling, and protein production in the endoplasmic reticulum. pDC activation predominantly “turned on” the chromatin regions associated with TF genes. Our in silico analyses pointed at the AP-1 family of TFs as less noticed but possibly important players in these cells after activation. AP-1 family members exhibit (1) increased gene expression, (2) enhanced chromatin accessibility in their promoter region, and (3) a TF DNA binding motif that is globally enriched in genomic regions that were found more accessible in pDCs after TLR9 activation.

**Conclusions:**

In this study we define the complete set of TLR9-regulated TFs in pDCs. Further, this study identifies the AP-1 family of TFs as potentially important but so far less well characterized regulators of pDC function.

**Supplementary Information:**

The online version contains supplementary material available at 10.1186/s12863-021-00991-2.

## Background

Transcription factors (TFs) are known to bind to DNA-regulatory sequences to either enhance or inhibit gene transcription during cell differentiation, at steady state, and for exertion of cell effector functions [1–3]. TFs also show unique expression patterns for different cell types and cellular states. The differentiation of distinct cell types from pluripotent stem cells is enabled by the expression of cell fate-determining TFs in progenitor cells. Transcription factors not only regulate cell development and effector functions by binding to *cis*-regulatory elements but also impact the accessibility of chromatin in different cell states [4]. These latter TFs are called pioneering TFs and have the ability to remodel chromatin and thus modify the epigenome [5]. Chromatin is dynamically modified during cell differentiation leading to a cell-type specific landscape [6, 7], which may be altered after cell activation. This process changes DNA accessibility for a particular set of TFs, that in turn modulate the expression of other genes important for cell identity and function. Efforts have been made to list and integrate all known mouse TFs in dedicated databases (db), such as Riken mouse TFdb [8] and TFCat [9], amongst others. However, most of these were built before 2010 and have not been updated. The AnimalTFDB, most recently updated in 2019, classifies the mouse TF reservoir based on the structure of the DNA binding domains [10, 11]. This database provides an accurate TF family assignment combined with TF binding site information in 22 animal species which also allows insight into TF evolution.

Plasmacytoid dendritic cells (pDCs) comprise a rare population of 0.2 to 0.8% of peripheral blood mononuclear cells [12]. They were first described more than 40 years ago as natural interferon (IFN)-producing cells (IPCS) that activate NK cells after virus recognition [13]. As we and others have shown, pDCs are now known for their capacity to produce large amounts of type I IFN in response to stimulation of their toll like receptors (TLRs) [14–18]. Ito et al. showed for example that IFNα/β transcripts produced by human pDCs after viral activation account for an unmatched 50% of all mRNAs in the cell [19]. In contrast to other dendritic cell (DC) subsets, pDCs express only a limited repertoire of TLRs, namely predominantly TLR7 and TLR9 [20], which recognize guanosine- and uridine-rich ssRNA and DNA containing CpG motifs [21–23]. After TLR7 and TLR9 activation, in addition to type I IFN production, pDCs acquire the ability to prime T cell responses [24]. CpG can be considered as an optimal and specific microbial stimulus for pDCs which induces TLR9 mediated signaling that leads to activation of IRF7 and NF-kB signaling pathways [25]. With regard to immunopathologies, unremitting production of type I IFN by pDCs has been reported in autoimmune diseases like systemic lupus erythematosus [26]. Moreover, when recruited to the tumor microenvironment pDCs may induce immune tolerance and thus contribute to tumor progression [27, 28]. Thus, exploiting CpG for immunotherapeutic treatment to both enhance and repress pDC responses to mediate antitumor activity [29], treat allergy [30], and autoimmunity [31] has been attempted in recent years. In addition, targeting specific TFs with the aim to control immunity and autoimmune disease [32] or to enhance cancer gene therapy [33] has become the focus of attention in recent decades to develop immunomodulatory drugs.

Over the last years, different TFs have been determined as cell fate-instructive TFs in DCs. In particular, absence of the interferon regulatory factor 8 (IRF8) resulted in pDC-deficient mice [34, 35]. Bornstein et al. further identified IRF8 as an inducer of cell-specific chromatin changes in thousands of pDC enhancers [36]. Further, mice deficient in the Ets family transcription factor Spi-B showed decreased pDC numbers in the bone marrow (BM) while pDC numbers were increased in the periphery. This indicated an involvement of Spi-B in pDC development, caused by a defective retainment of mature nondividing pDCs in the BM [37]. In contrast to the phenotype of *Spi-B*-deficient mice, *Runx2*-deficient animals exhibited normal pDC development in the BM but reduced pDC numbers in the periphery due to a reduced egress of mature pDCs from the BM into the circulation [38, 39]. Finally, the *Tcf4*-encoded TF E2–2 is essentially required for pDC development as either its constitutive or inducible deletion in mice blocked pDC differentiation [40]. Using a combined approach to evaluate genome-wide expression and epigenetic marks a regulatory circuitry for pDC commitment within the overall DC subset specification has been devised [41]. Even though the functions of selected cell fate TFs have been well described in pDCs, to our knowledge no global TF expression analysis for early timepoints after pDC activation has been performed for this cell type. Of note, a first large-scale analysis of the chromatin landscape in primary human cDCs and pDCs after 48 h of TLR7 activation leading to type I IFN induction was recently performed by Leylek et al. [42]. As an alternative stimulus CD40L was used where no induction of type I IFN was observed. The authors found that pDCs undergo large-scale chromatin changes that were stimulus dependent, and that their conversion into cDC-like cells follows a linear trajectory. They correlated the pDC chromatin landscape with protein and RNA expression of pDC cell fate factors, as well as TF activity. Their study represents a first in-depth overview of TF expression and activity in human primary pDCs at 48 h after TLR7 stimulation, representing a late phase of activation. In the study presented here we performed a global TF analysis at 2 h, 6 h, and 12 h after TLR9 activation. These represent early events after virus infection during which pDCs are known for their capacity to produce copious amounts of type I IFN. Thus, our analyses give insights into the dynamics of TF activity early upon activation of mouse pDCs.

In the present study, we performed a detailed analysis on the changes in expression and chromatin accessibility for the complete set of all known TFs in pDCs in an early time course after activation. To this purpose, we used the AnimalTFDB data base and combined RNA-Seq, ATAC-Seq, and Gene Ontology analyses to define global TF gene expression, chromatin landscapes, and biological pathways in pDCs following activation. We defined epigenetic and transcriptional states using purified murine BM-derived Flt3-L cultured pDCs 2 h, 6 h, and 12 h after TLR9 activation as compared to steady state. Based on our findings, we suggest a novel set of CpG-dependent TFs associated with pDC activation. We further identify the AP-1 family of TFs, which are so far less well characterized in pDC biology, as novel and possibly important players in these cells after activation.

## Results

### Expression of transcription factors in naïve and activated pDCs

**Fig. 1:**
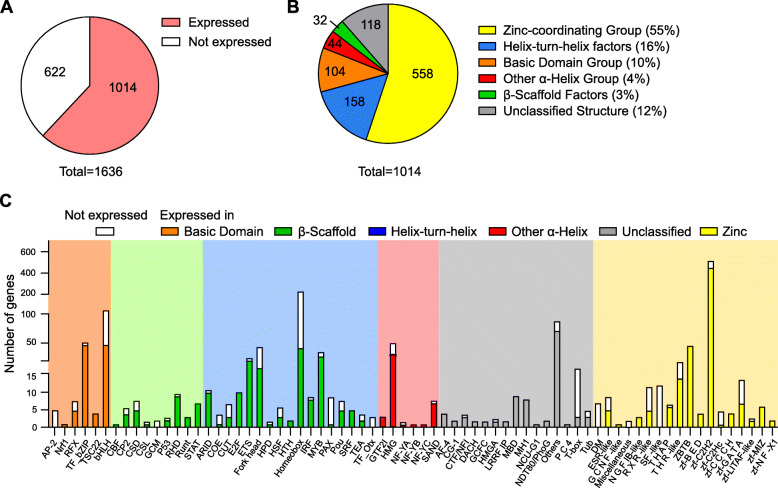
Expression of transcription factors in pDCs. **A** Expression of TFs in pDCs in at least one of the following conditions: naïve, CpG 2 h, 6 h or 12 h (*n* = 3 per condition). **B** Categorization of the expressed TFs according to Hu et al. [11]. **C** Number of expressed vs non-expressed genes per TF family of a TF class is plotted

To assess the impact of pDC activation on global TF expression in these cells, we simulated early events after virus infection in a time course study. To this end, we performed RNA-Seq of sorted BM-derived Flt3-L pDCs from C57BL/6N mice that were either left untreated or stimulated with CpG for 2 h, 6 h, or 12 h. This synthetic double-stranded DNA specifically activates endosomal TLR9 and is known to induce a robust type I IFN production [18]. As the global definition of the mouse TF reservoir in this study we used 1636 genes annotated by Hu et al. as TFs in the mouse genome [11]. We evaluated the expression of all TFs in pDCs according to a formula by Chen et al.*,* which takes into consideration the library length of the RNA-Seq run and the gene length to determine whether the gene is expressed or not [43]. We found that 1014 TFs (70% of all annotated TFs) are expressed in at least one condition, naïve or after TLR9 activation (2 h, 6 h, 12 h) (Fig. 1A). The TFs expressed in pDCs were allocated to the different TF classes based on their DNA binding domain as described in the AnimalTFDB [11] (Fig. 1B). We found that more than half of all TFs (55%, 558 TFs in total) expressed in pDCs belong to the Zinc-coordinating TF group which use zinc ions to stabilize its folding and classically consist of two-stranded β-sheets and a short α-helix. Helix-turn-helix factors, of which 158 (16%) were expressed in pDCs under the defined conditions, comprise several helices mediating multiple functions such as insertion into a major DNA groove, stabilization of the backbone and binding to the overall structure of the DNA [44]. Furthermore, 10% (104 TFs) of all TFs expressed in pDCs belong to the Basic Domain group, which contains TFs that become α-helically folded upon DNA binding [45, 46]. Forty-four expressed TFs (4%) belong to the Other α-Helix group exhibiting α-helically structured interfaces are required for DNA binding. In addition, 32 of the TFs (3%) found in pDCs are β-Scaffold factors which use a large β-sheet surface to recognize DNA by binding in the minor groove. Lastly, another ~ 100 TFs (12%) were of unclassified structure, meaning their mode of action for DNA binding is unknown. Strikingly, some TF families were not expressed in pDCs at all (Fig. 1C), such as the AP-2 family in the Basic Domain group, the GCM family in the β-Scaffold group, the Orthodenticle homeobox (Otx) TFs in the Helix-turn-helix group, Steroidgenic factor (SF)-like factors in the Zinc-coordinating group, and the DM group, first discovered in *Drosophila melanogaster*, among the unclassified TFs. Other TF families showed expression of all family members in at least one condition (steady state, or CpG 2 h, 6 h, 12 h), such as the Transforming growth factor-β stimulated clone-22 (TSC22) family in the Basic Domain group, Runt and Signal Transducers and Activators of Transcription (STAT) factors from the β-scaffold classification, and E2F and Serum response factor (SRF) factors in the Helix-turn-helix group. In summary, 70% of all genes annotated as TFs in the mouse genome (1014 out of 1636) were expressed either in naïve or activated pDCs (CpG 2 h, 6 h, 12 h), covering a wide range of TF classes based on different DNA binding mechanisms.

### Activation-dependent TF expression changes

**Fig. 2:**
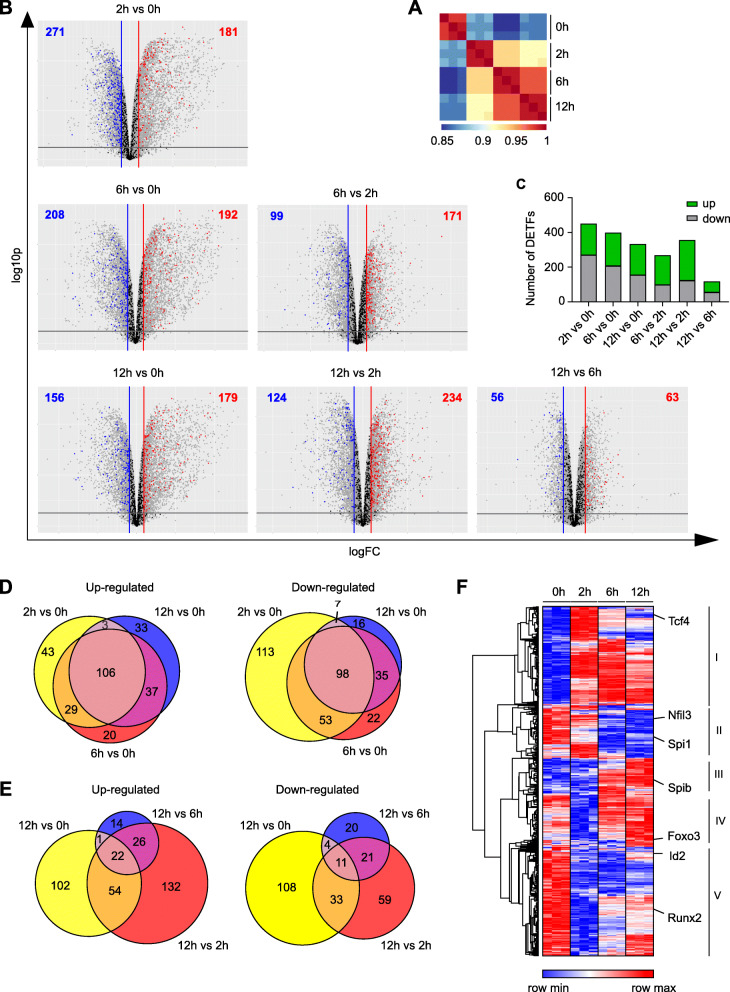
RNA-Seq reveals significant TF expression changes after pDC activation. **A** Pearson correlation plot for samples used in RNA-Seq. pDCs (CD3^−^CD19^−^CD11c^+^CD11b^low^B220^+^SiglecH^+^CD317^+^) were sorted from BM-derived Flt3-L cultures of C57BL/6 N mice and cells were left either naïve or stimulated with CpG for 2 h, 6 h or 12 h. **B** Volcano plots showing global expression of genes in sorted pDCs at steady state and after 2 h, 6 h, and 12 h of CpG stimulation. TF genes with a |FC| > 2 and a *p*-value of < 0.05 corrected for the false discovery rate (FDR) were considered significantly differentially expressed and are marked in colour (red and blue). **C** Bar chart depicting number of DETFs that are up or down-regulated between the respective conditions (|FC| > 2, *p* < 0.05). **D, E** Venn diagrams displaying significantly up and down-regulated TF genes (*p* ≤ 0.05, |FC| ≥ 2) between stimulated pDCs vs naïve pDCs (**D**) and 12 h pDCs stimulated vs naïve, 2 h and 6 h stimulated pDCs (**E**). **F** Heatmap showing normalized expression values (cpm, count per million) of differentially expressed TF genes from (**B**) in pDCs at steady state and after 2 h, 6 h, and 12 h of CpG stimulation. Hierarchical clustering on rows with average linkage and the One minus Pearson correlation metric was performed

We next investigated the impact of pDC activation on changes in expression of TFs using our time course RNA-Seq study. The similarity of our biological replicates in each condition was evaluated with a Pearson correlation analysis. Our results revealed high similarity (< 95%) for the biological replicates used in the respective conditions of the RNA-Seq data set. Notably, the differences in the Pearson correlation coefficient between the naïve and first stimulation time point (CpG 2 h) were higher than the differences observed between the later CpG stimulation time points (6 h, 12 h) (Fig. 2A). We used the data for differential expression analysis of genes between pDC states, not only comparing TF expression levels between different CpG stimulation time points vs steady state but also between the different CpG stimulation time points between each other (Fig. 2B). The total number of differentially expressed TFs (DETFs) with a fold change |FC| > 2 and a *p* < 0.05 between stimulated vs naïve pDCs (452 DETFs in 2 h vs 0 h; 400 DETFs in 6 h vs 0 h; 335 DETFs in 12 h vs 0 h) was higher than the absolute number of TFs showing expression changes between the CpG conditions (270 DETFs in 6 h vs 2 h; 119 DETFs in 12 h vs 6 h; 358 DETFs in 12 h vs 2 h). This reflects the results from the Pearson correlation analysis (Fig. 2A). Interestingly, by comparing TF gene expression in 2 h stimulated vs unstimulated pDCs, a higher number of TF genes were down-regulated in expression after TLR9 stimulation than were upregulated in these cells (271 vs 181). With increased duration of pDC stimulation, the difference in the number of TFs that were up- vs down-regulated diminished (208 down vs 192 up in 6 h vs 0 h). Finally, at the longest stimulation time used in this study (12 h vs 0 h), the number of up-regulated TF genes was higher than the number of down-regulated TF genes (179 vs 156). Comparing the CpG stimulated samples amongst each other, more TFs exhibited increased expression with longer stimulation times than there were TFs showing reduced expression levels (171 up vs 99 down in 6 h vs 2 h; 63 up vs 56 down in 12 h vs 6 h; 234 up vs 124 down in 12 h vs 2 h) (Fig. 2B and C). Searching for hints on the dynamics of TF activity upon TLR9 activation of pDCs we defined a set of early-responding up and down-regulated TFs (181 and 271 genes, respectively; 2 h vs 0 h, FC ≥ 2, *p* ≤ 0.05), and late-responding up and down-regulated TFs (228 and 231 genes, respectively; 6 h vs 0 h and 12 h vs 0 h, FC ≥ 2, p ≤ 0.05). In a first analysis, motifs of immediate TLR9 signaling dependent TFs (IRF3, IRF7, NFkB1, NFkB2) were screened for occurrence among the promoter regions (− 1000 of TSS) of early-responding TF genes. We found 308 and 360 significant motif hits (*p* < 0.0001) in the promoter sequences of early-responding TF genes that were up or down-regulated, respectively. The significant motif hits occurred with a similar frequency for IRF3, IRF7, NFkB1 and NFkB2 ranging from 69 to 87 and 69–114 for up and down-regulated TF genes, respectively (Tables S1, S2). This suggests that early signaling TFs both initiate and repress expression of TFs by binding onto the respective promoter regions. While the gene expression inducing function of IRFs and NFkBs is well known, a repressing function has only been reported a few times. NFkB has been shown to mediate transcriptional repression of the hormone gastrin [47], and IRFs such as IRF3 have been shown to mediate inhibition of IFNA4 through binding to its promoter [48]. In a second analysis, we evaluated the possible activation of late-responding TF genes by the higher expressed early-responding TF genes. To this end, the known TF motifs from the HOCOMOCO database for the early-responding TFs were used for FIMO analysis to examine their occurrence among the promoter regions (− 1000 of TSS) of the late-responding genes. We determined 1027 and 1277 significant motif occurrences (FDR-corrected *p*-value< 0.05) for up and down-regulated late-responding TF genes, respectively. Interestingly, two TF motifs dominated among late-responding TF genes, namely KLF6 and STAT1 that appear 768 and 245 times for up-regulated genes and 649 and 341 times for down-regulated late-responding TF genes, respectively (Tables S3, S4). This suggests that KLF6 and STAT1 bind extensively to promoter regions of TF genes that are both up and down-regulated at 6 h and 12 h after TLR9 activation of pDCs. Possibly, KLF6 and STAT1 binding has both activating and inhibiting effects. KLF6 has been reported to regulate the expression of various genes and has been associated with both wound healing processes and suppression of tumorigenesis [49]. STAT1 is activated through receptor-Jak tyrosine kinase signaling cascades, which are activated by cytokines such as type I IFNs [50]. While it is known that STAT1 is required for ISG induction upon virus infection of mice [51], we, to our knowledge, are the first to describe the enrichment of the STAT1 DNA binding motif among promoter regions of late-responding genes upon CpG activation of pDCs. Notably, a few additional TFs showed motif occurrence only among the down-regulated late-responding TF genes, for example ARNT2 and PRDM1 that appeared 13 and 6 times, respectively, hinting at a specific inhibitory role of these factors in pDC biology. ARNT2 has been shown to control gene expression through NCoR2-mediated repression [52], and PRDM1 is known to silence many genes including Pax5 and c-Myc [53]. Immediate TLR9 signaling dependent TFs (IRF3, IRF7, NFkB1, NFkB2), however, do not exert control of expression of late-responding TF genes as their DNA binding motif is not found among the promoter regions of late-responding TF genes, with the exception of NFkB2 and IRF5 that appeared once among the up and down-regulated late-responding TFs, respectively.

We next evaluated the overlap of up or down-regulated TF genes (*p* ≤ 0.05, |FC| ≥ 2) between stimulated pDCs vs naïve pDCs (Fig. 2D) and within the stimulation time course comparing 12 h pDCs stimulated vs naïve, 2 h and 6 h stimulated pDCs (Fig. 2E). Our results show that approximately 100 TF genes overlap in all conditions comparing significantly up-regulated  (106 genes) or down-regulated genes  (98 genes), respectively, of stimulated (2 h, 6 h, 12 h) vs naïve pDCs. Further, we found that the highest number of overlapping genes exist in the 12 h vs naïve and 6 h vs naïve comparisons for both up and down-regulated genes (Fig. 2D). The analyses comparing the 12 h stimulation timepoint vs naïve, 2 h and 6 h stimulation shows an overlap of only 22 up- and 11 down-regulated TF genes (Fig. 2E). Here, the biggest overlap of genes is found between 12 h vs 6 h and 12 h vs 2 h stimulated pDCs. Thus, the effect of CpG stimulation on the expression of TF genes in pDCs shows the highest similarity for 6 h and 12 h stimulated pDCs compared to naïve pDCs. In total, we identified 661 unique TF genes that are differentially expressed between at least one of the compared pDC states (|FC| > 2, *p* < 0.05, pDC at steady state, or after CpG activation at 2 h, 6 h, 12 h). To evaluate patterns of expression changes for all 661 differentially expressed TFs, we next carried out hierarchical clustering of all TF genes based on the normalized expression in naïve and stimulated pDCs (Fig. 2B). This led to the definition of five different clusters of TFs according to their expression pattern (Fig. 2F). Cluster I, IV and V contained TFs with large expression changes after short duration of pDC stimulation (2 h), while cluster II and III contained TFs that exhibit altered expression only with longer duration of cell stimulation (6 h, 12 h). Cluster V contained genes that were all down-regulated at any time point after CpG stimulation as compared to the unstimulated condition (Fig. 2D). In more detail, TFs driving either pDC (e.g. *Tcf4, Spib, Runx2*) or classical DC (cDC) (e.g. *Nfil3, Spi1, Id2*) development [34–38] were distributed over all clusters I to V. This highlights variable expression patterns of DC cell fate TFs after pDC activation. In summary, in this time course study that models early events after virus infection, we identified in total 661 unique CpG-dependent TF genes that show significant differential expression in at least one condition compared to another (|FC| > 2, *p* < 0.05, pDC at steady state, or after CpG activation at 2 h, 6 h, 12 h). Further, pDC activation showed time dependent activating as well as inhibiting effects on the expression of TFs.

### Gene ontology analysis of CpG-dependent TFs

**Fig. 3:**
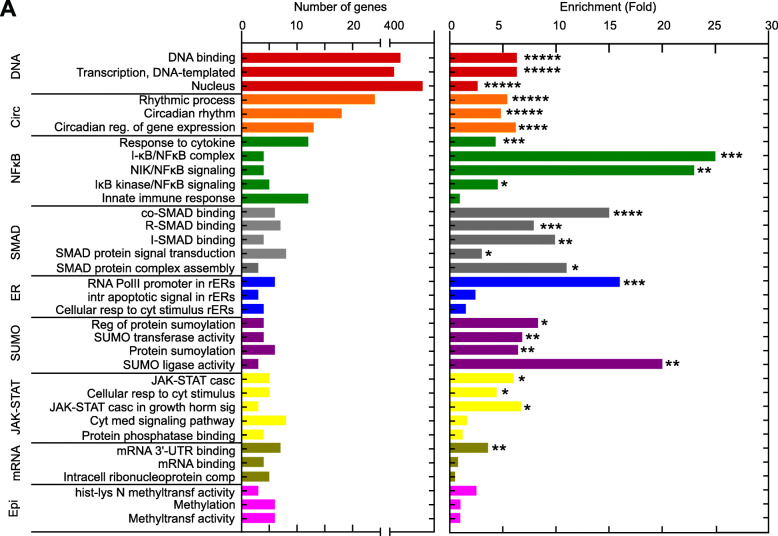
Gene Ontology analysis of CpG-dependent TFs. 661 CpG-dependent TFs (|FC| > 2, p < 0.05) were analysed by DAVID functional annotation to produce gene clusters (> 2 genes/cluster) corresponding to biological process (BP), molecular function (MF), and cellular component (CC) GO annotation terms. Those significantly associated with the TF gene list are plotted with the numbers of genes for each term along with the fold enrichment for each term. A few terms were excluded as being redundant or having wider meaning (Table S5). Abbreviations are as follows: casc = cascade; cyt = cytokine; horm = hormone; med = mediated; reg = regulation; rERs = response to endoplasmic reticulum stress; resp. = response; sig = signaling

Next, downstream gene ontology (GO) analyses of RNA-Seq data were performed to unravel the biological processes in which CpG-dependent TFs are involved. For this purpose, functional annotation clustering with the 661 TF encoding genes defined as CpG-dependent (|FC| > 2, p < 0.05) was performed on DAVID including terms for biological processes (BP), molecular functions (MF), and cellular components (CC). The analysis produced 16 clusters, out of which the 9 non-redundant and most relevant in the context of innate immunity are depicted in Fig. 3A (complete list in Table S5). The GO analyses produced an individual fold enrichment for each GO term (Fig. 3A, right column), and in addition, an enrichment score for each cluster containing several GO terms (Table S5). The order of the clusters from top to bottom follows a decrease in the cluster enrichment score, establishing a hierarchy of importance for the biological processes affected. Cluster one contained GO terms for DNA binding, transcription, and nuclear localization with a ~ 5 fold enrichment comprising more than 400 genes in each term. This confirmed the inherent DNA binding capacity of the defined murine TF reservoir by Hu et al. [11] and proved the applicability of our approach. The following clusters comprised less than 25 unique genes per GO term but significant fold enrichments for most GO terms drawing attention to specific TFs involved in particular biological processes in pDC activation. Cluster 2 contained GO terms associated with the circadian rhythm and regulation of gene expression (e.g. *Klf10, Jun*). We further found GO terms enriched for the IκB/NFκB complex, NIK/NFκB signaling, and IκB kinase/NFκB signaling (e.g. *Nfkb1, Nfkb2, Rel*), which showed the highest fold enrichment (up to 25 fold) among all GO terms and clusters. In line with this, it is well known that CpG activates the canonical TLR9-Myd88-NFκB/IRF7 signaling pathway in pDCs [54]. Another cluster contained processes involving SMAD proteins (e.g. *Smad1, Smad2, Smad3*), signal transducers for TGFβ receptors, involved in receptor binding, signal transduction, and protein complex assembly. Of note, it is known that pDCs exposed to TGFβ lose their ability to produce type I IFN after TLR9 stimulation [55], which may be due to the reported conversion of pDCs into cDCs upon TGFβ and SMAD3 protein exposure [56, 57]. Another significantly enriched cluster comprised GO terms for various processes involving the endoplasmic reticulum (e.g. *Cebpb, Ddit3*), an important site of intracellular protein and lipid assembly. GO terms containing TFs that regulate sumoylation (e.g. *Pias4, Egr2*), posttranslational modifications that e.g. coordinate the repression of inflammatory gene expression during innate sensing [58], were also significantly enriched and clustered together. As expected, CpG-dependent TFs were enriched in GO terms for the JAK-STAT signaling pathway (e.g. *Stat1, Stat2, Stat3*) activated by binding of type I IFN to the type I IFN receptor. TFs affecting mRNA binding processes (e.g. *Mbd2, Ybx2*) which are required for synthesizing proteins at the ribosomes, were also affected. The fact that epigenetic modulators (e.g. *Prdm9, Kmt2c*) were enriched highlights the importance of gene expression regulation of TFs in pDCs by modifications that alter the physical structure of the DNA after CpG stimulation. In summary, we found that CpG-dependent TFs are involved in a wide variety of biological processes, such as circadian regulation, mRNA binding, and signaling pathways such as the NFκB and JAK-STAT pathways. The analyses revealed the importance of these biological processes being affected by pDC activation in a hierarchical manner according to their attributed relevance. This opens up the opportunity to investigate specific TFs involved in processes that have not been fully elucidated for pDC biology.

### pDC activation modulates chromatin accessibility for binding of TF families

**Fig. 4:**
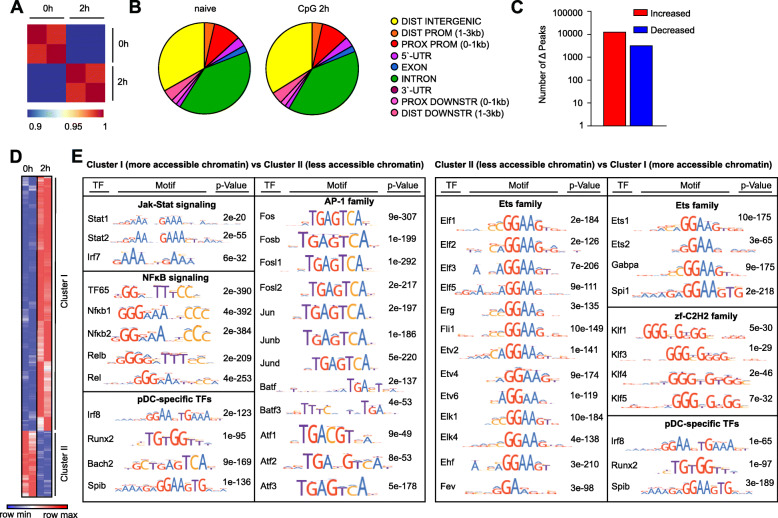
pDC activation increases and decreases chromatin accessibility of thousands of regions. **A** Pearson correlation plot for samples used in ATAC-Seq. pDCs (CD3^−^CD19^−^CD11c^+^CD11b^low^B220^+^SiglecH^+^CD317^+^) were sorted from BM-derived Flt3-L cultures of C57BL/6 Nmice and cells were left either naïve or stimulated with CpG for 2 h (*n* = 2). **B** Genomic location distribution of open chromatin sites in naïve and CpG stimulated pDCs according to ATAC-Seq. Two biological replicates were used per condition, and results are shown for pooled samples per condition. **C** Number of differentially accessible peaks detected using DESeq2, comparing naïve to 2 h CpG stimulated pDCs, |FC| > 2 and *p* < 0.05. **D** Heatmap of normalized ATAC-Seq peak intensities (log_2_FC relative to the mean for each peak). Limited to peaks (16,607) that are condition-dependent with |FC| > 2 and p < 0.05 for at least one pairwise comparison of interest. **E** Differential motif analysis for cluster I and II from (**D**) using MEME Centrimo and the HOCOMOCO v11 motif database. Significant motifs were categorized into known TF families for visualization and interpretation

Another hallmark of cell activation is the modification of the chromatin landscape. To better understand how the chromatin accessibility of different TF families is altered in pDCs in the course of activation, we performed ATAC-Seq in naïve and 2 h CpG activated pDCs. Pearson correlation analysis for the ATAC-Seq data reveals > 95% similarity for all biological replicates (Fig. 4). A quantitative analysis of peak intensities across experimental conditions and a differential analysis to determine the number of activation-dependent accessible chromatin peaks was performed. In addition, the qualitative distribution of ATAC-Seq peaks based on their genomic location (such as introns, 3′-UTRs, distal (1–3 kb) and proximal (0-1 kb) promoter regions) was determined. Comparing the specific genomic locations with accessible chromatin between naïve and 2 h CpG stimulated pDCs, we found that chromatin is mostly open in distal intergenic and intron regions in both conditions. However, there was no apparent shift in the distribution of genomic locations where chromatin is accessible in pDCs after cell activation (Fig. 4B). This suggests that TLR9 activation regulates the chromatin accessibility globally in pDCs but does not induce shifts in the distribution of genomic locations in the in the chromatin landscape per se. Overall, we detected ~ 116,000 accessible regions (peaks) across samples in naïve and activated states. Next, we performed a differential analysis using the DESeq2 algorithm to quantify the number of CpG-dependent accessible peaks. pDC activation substantially altered the chromatin landscape leading to ~ 16,600 altered accessible regions (|FC| > 2, *p* < 0.05, Fig. 4C, D). In detail, 2 h CpG stimulation of pDCs resulted in 13,226 peaks with increased accessibility and 3381 peaks with decreased accessibility (Fig. 4C, D). Roughly 80% of all CpG-dependent chromatin regions in 2 h stimulated pDCs exhibited increased DNA accessibility as compared to naïve pDCs. This suggests that more of the pDC chromatin landscape is "turned on“ rather than being "turned off“ after pDC activation. A global gain of ATAC-Seq signal is usually associated with increased gene expression as increased DNA accessibility allows TF to bind to DNA-regulating elements to induce expression. Investigating TF expression, we found an up-regulation of 181 TF genes and a down-regulation of 271 TF genes comparing 2 h stimulated pDCs with naïve pDCs (Fig. 2B). Thus, surprisingly, there were more down-regulated than up-regulated TF genes. There might be several explanations for our observation. For one, with an increasing duration of pDC stimulation we observed a shift from more TF genes being down-regulated in expression (2 h vs 0 h) toward more TF genes being up-regulated (12 h vs 0 h). The observed gain of chromatin accessibility in 2 h stimulated vs naïve pDCs may facilitate the binding of TFs to the DNA which then induce an increased expression of TF genes that only becomes visible in gene expression within later hours at 6 h and 12 h. Second, TF binding can also inhibit expression. On an individual gene level, we determined a strong correlation between more open chromatin and increased gene expression, and vice versa. In detail, 150 genes had at least one more open chromatin site after TLR9 stimulation, and out of these 111 genes exhibited also higher expression levels. On the other hand 29 genes contained at least one chromatin site that was less accessible after TLR9 stimulation out of which 12 genes were also less expressed. In addition, we identified 14 genes that fulfilled two criteria (a) decreased expression, and (b) increased ATAC-Seq signal at 2 h: Cebpa, Elmsan1, Foxk1, Foxo1, Irf4, Mta3, Nfatc2, Pias2, Rere, Runx2, Smad6, Trerf1, Zbtb38, Zfp592. The majority of these factors were still significantly down-regulated at 6 h and 12 h after CpG stimulation as compared to naïve pDCs. Only a minority of factors (Foxk1, Foxo1, Zbtb38 Zfp562) showed an increase in expression at 6 h and 12 h after TLR9 activation compared to expression levels in naïve pDCs. We conclude from this analysis that an increase in ATAC-Seq signal at 2 h after CpG stimulation does not result in increased expression of the majority of these TF genes at 6 h and 12 h. Rather the more accessible chromatin may facilitate inhibitory TF binding resulting in repression of gene expression for these TFs. Interestingly, one TF family with significant motif enrichment among the DNA stretches with increased chromatin accessibility is e.g. the AP-1 family of TFs. This family contains TFs without a DNA transactivation domain such as the BATF factors which are known to rather inhibit gene expression.

To unravel the biological significance of the activation-dependent chromatin states for the more accessible (Cluster I, Fig. 4D) vs the less accessible (Cluster II, Fig. 4D) DNA regions in pDCs, a differential motif analysis using the HOCOMOCO database [59] was performed (Fig. 4E). The purpose of the analysis was to identify TF families that gain or lose access to DNA after pDC activation which would hint at pathways being affected after activation. At the same time, this unbiased approach allows the identification of TFs that have not been associated with this cell type before. This motif analysis revealed that TFs belonging to the JAK-STAT and the NFκB signaling pathway have increased accessibility to their specific DNA binding regions after CpG stimulation. Besides the NFκB family, we identified the AP-1 family of TFs as one of the most significant hits to gain access to the DNA in our search. This type of TF remains so far less well characterized in pDCs after pathogen encounter or in pDC-specific functions in chronic inflammatory or autoimmune disorders. Albeit the AP-1 member c-Fos has been shown to be required for type I IFN induction, a hallmark function of pDCs, in osteoclast precursor cells after RANKL treatment [60]. On the other hand, Ets family members belonging to the Helix-turn-helix family of TFs and Zinc-coordinating zf-C2H2 TFs had less access to DNA. Strikingly, pDC-driving cell fate TFs such as IRF8 and RUNX2 showed motif enrichment in two sets of regions, one set with increased and another set with decreased chromatin accessibility after pDC activation. Hence, pDC-driving cell fate TFs both gained and lost access to specific DNA regions after TLR9 activation. We next performed a more detailed analysis searching for enrichment of TF motifs among all regions that contain the promoter sequence of one or more genes. As TFs can regulate gene expression by binding to the promoter site of genes this analysis hints at TF families that exert a functional binding occupancy in the investigated chromatin regions. We previously determined that 13,226 regions exhibit increased chromatin accessibility after pDC activation. Out of these, 2174 regions were associated with the promoter of one or more genes. An unbiased motif enrichment search revealed that TFs belonging to the NFκB family (e.g. NFκB1, NFκB2, TF65), the AP-1 family (e.g. ATF3, JUN, FOSB), and the JAK-STAT family (e.g. STAT1, STAT2), as well as pDC cell fate TFs (e.g. RUNX2, IRF8) are among the top hits for TFs with DNA binding domains present in promoter associated chromatin regions which gain accessibility after pDC activation (Table S6). In summary, the differences in chromatin landscapes of naïve and 2 h CpG stimulated pDCs point to a substantial amount of epigenetic modulation of thousands of pDC regions. Also, these analyses unravelled the AP-1 family of TFs, which have so far been less well characterized in pDC biology, as possibly important players in these cells after activation.

### TFs show activation-dependent expression and chromatin accessibility

**Fig. 5:**
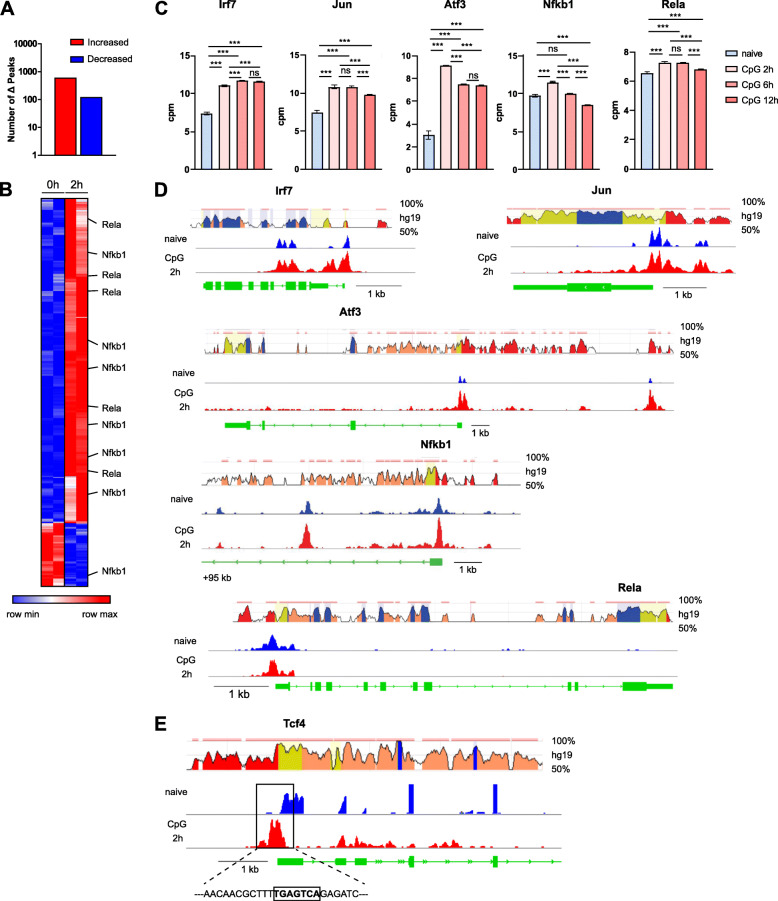
TFs show CpG-dependent expression and chromatin accessibility. **A** Number of differentially accessible peaks of genomic regions associated with TF genes detected using DESeq2 comparing naïve to 2 h CpG stimulated pDCs, |FC| > 2 and *p* < 0.05. **B** Heatmap of normalized ATAC-Seq peak intensities (log_2_FC relative to the mean for each peak) limited to 540 peaks from (**A**) that are condition-dependent with |FC| > 2 and p < 0.05 for at least one pairwise comparison of interest. **C** The bar graph depicts normalized expression values obtained from RNA-Seq and statistics calculated with edgeR. **D, E** Top panel presents screen shots from the ECR (evolutionary conserved regions) Browser web site of the respective indicated gene. Exonic regions are shown in blue, intronic regions in pink, UTRs in yellow, and CNS in red. Bottom panels present ATAC-Seq peaks in naïve and CpG stimulated (2 h) pDCs for the indicated genes visualized with IGV. The AP-1 motif within the promoter sequence of the *Tcf4* gene is highlighted in (**E**)

As shown above, pDC activation results in significant alterations of the chromatin landscape in pDCs making the DNA more or less accessible to specific TF families on a global level. We next analysed the impact of pDC activation on regions associated with TF genes themselves by evaluating regions ranging from 1 kb upstream of the transcriptional start site (TSS) to 1 kb downstream of the poly adenylation site. pDC activation altered the chromatin landscape of ~ 750 accessible regions associated with TF genes (|FC| > 2, *p* < 0.05, Fig. 5A). In detail, 2 h stimulation of pDCs resulted in 627 peaks with increased accessibility and 126 peaks with decreased accessibility to regions associated with TF genes (Fig. 5A). 83% of all CpG-dependent chromatin regions in 2 h stimulated pDCs exhibited increased DNA accessibility as compared to naïve pDCs. Looking at the complete genome, pDC activation mostly “turned on” the chromatin landscape. This correlated well with the state of chromatin regions associated with TF genes themselves. These are as expected also mostly “turned on”, indicating that the TF genes follow the general direction of chromatin change. Finally, an integrative approach using the RNA-Seq and ATAC-Seq data was conducted analysing the differential chromatin states of regions associated with differentially expressed TF genes. This revealed 540 TF regions out of the overall ~ 750 chromatin regions that are significantly associated with a differential RNA expression of the respective TF gene (Fig. 5B). Out of these chromatin peaks we found 209 unique TF genes being associated with the differentially opened chromatin regions. Thus, pDC activation modulates the chromatin of most genes in more than one region associated with the respective gene, as shown here for the NFκB family members *Nfkb1* and *Rela* (Fig. 5B). Interestingly 92 TFs showed both higher expression and higher chromatin accessibility at 2 h after CpG stimulation as compared to steady state. A motif search for immediate TLR9 signaling-dependent TFs revealed that the IRF3, IRF7, NFkB1 and NFkB2 motifs occurred at a similar frequency ranging from 34 to 49 times among the promoter regions (− 1000 of TSS) of early-responding TF genes with increased expression and chromatin accessibility (Table S7). To identify potential novel players in pDC biology after cell activation, we integrated the results of our motif analysis, the RNA expression levels, and chromatin states for all TFs. We focused our search on factors that fulfil the following criteria after pDC stimulation: (i) increased gene expression, (ii) enhanced chromatin accessibility, and (iii) enriched TF DNA binding motif in the genomic regions that are more accessible. Mining our dataset, we found that TFs already known to be important in TLR9-mediated signaling such as IRF and NFκB TFs met the requirement as expected. Additionally, members of the AP-1 family such as ATF3 and JUN, which received little mention for pDC biology in literature so far, also fulfilled these criteria. The candidates of all three families exhibited a significantly increased mRNA expression 2 h after pDC activation as compared to naïve pDCs. At 6 h after stimulation, expression remained at the same level (Jun, Rela), increased further (Irf7) or decreased (Atf3, Nfkb1). After 12 h pDC stimulation, expression remained at the same level (Irf7, Atf3) or even decreased (Jun, Nfkb1, Rela) (Fig. 5C). In line with an increased expression of the selected TFs 2 h after cell activation as compared to the naïve state, we found an increased accessibility of chromatin in the proximal promoter region of the *Irf7*, *Jun*, *Atf3*, *Nfkb1,* and *Rela* genes. Two regions of the *Nfkb1* gene, one proximal and another distal from the TSS of the gene, indicated increased DNA accessibility after CpG stimulation at 2 h as compared to the naïve condition. While *Atf3*, *Nfkb1* and *Rela* are characterized by single or a small number of open chromatin peaks, several peaks in the *Irf7* and *Jun* gene were found, both proximal and after the TSS in the intergenic region. Of note, the core structural elements regulating gene expression for the proximal promoter and the intergenic regions were well conserved between mouse and human for all newly identified candidates (top panels, Fig. 5D). The potential relevance of the AP-1 factors for pDC biology was further investigated by searching for the common AP-1 motif (TGA[G/C]TCA) [61] among all open chromatin regions associated with pDC driving TF genes (*Runx2, Tcf4, Spib, Irf8, Bcl11a*). Using the MEME-FIMO search tool, we found an AP-1 motif in the proximal promoter site of the *Tcf4* gene which encodes the E2–2 protein (Fig. 5E). As AP-1 has not been implicated so far in E2–2 gene regulation this finding warrants further investigation. In summary, we found that pDC activation mostly “turns on” TF genes resulting in significant expression changes along with more accessible DNA in promoter and or intergenic regions. Moreover, we newly identified the AP-1 family as a set of TFs associated with pDC activation.

## Discussion

In this study we investigated the yet unknown global expression patterns of the TF reservoir of pDCs in in a time course after activation in combination with DNA accessibility analysis for implicated TF families. Combining RNA-Seq, ATAC-Seq, and GO analyses, we defined specific sets of TLR9-modulated TFs with known roles in pDC differentiation and function, but also TFs so far not implicated in pDC biology.

We used as the basis of our study the definition of the murine TF reservoir in the AnimalTFDB [11] and found that 70% of all genes annotated as TFs in the mouse genome (1014 out of 1636) were expressed in at least one condition, naïve or CpG-activated pDCs (2 h, 6 h, or 12 h). These covered a wide range of TF classes defined by their respective DNA binding mechanisms. Interestingly, some TF families showed expression of all family members. Among those, we found factors that have been shown to be of particular importance in pDC biology, such as Runx2 of the Runt family [38]. We further determined differential expression of TF genes in our time course study. To gain first insights about the dynamics of TF induction, we searched for motif occurrences in early (2 h) and late-responding (6 h, 12 h) TF genes. We found that the motifs of immediate signaling-dependent TFs such as IRF3, IRF7, NFκB1 and NFκB2 occur at similar frequencies among the promoter sequences of early-responding TF genes that are higher expressed and exhibit more accessible DNA at 2 h stimulation vs steady state condition. This result suggests that immediate TLR9 signaling-dependent TFs induce the expression of early-responding TF genes in pDCs. We further found that the induction of late-responding TF expression after TLR9 activation in pDCs may be orchestrated by a small number of TFs that exhibit increased expression at 2 h after pDC activation, such as KLF6 and STAT1, by binding onto the respective promoter sites. The analysis of shared gene expression changes between different pDC states revealed a high similarity of TF patterns between 6 h and 12 h stimulated pDCs, as compared to naïve pDCs, respectively. This result reflects the overall similarity of global expression patterns observed in the Pearson correlation analysis of the RNA-Seq data. Within the time course study, we observed the biggest overlap of up and down-regulated genes between the stimulated pDCs (2 h, 6 h, 12 h), stressing a pronounced shift in TF expression between the naïve and TLR9 activated state. Downstream GO analyses of RNA-Seq data allowed a biological classification of all TFs showing involvement in a wide variety of biological processes, such as the NFκB and JAK-STAT signaling. It has been well established that the production of type I IFN by pDCs upon TLR9 activation depends on the canonical TLR9-Myd88-NFκB/IRF7 signaling pathway [54]. In this regard, it has been reported that NFκB and cREL are key players in pDC differentiation and survival programs after TLR9 activation by CpG. *Nfkb1*^−/−^ *cRel*^−/−^ double knock-out pDCs were still able to produce type I IFN upon CpG administration but failed to produce IL-6 or IL-12 and did not acquire a dendritic phenotype but rather underwent apoptosis [62]. Here, we show for the first time the time-dependent patterns of gene expression for TFs involved in NFκB and JAK-STAT signaling upon pDC stimulation. Not only expression of these factors was enhanced in pDCs after CpG treatment, but also DNA binding sites for factors from the NFκB and JAK-STAT signaling pathways were identified as globally enriched in a differential motif analysis comparing regions with increased vs decreased chromatin accessibility. In addition, we found changed expression patterns of TFs important for circadian gene regulation in activated pDCs over time. In this regard, it has been reported that up to 10% of the transcriptome is under circadian regulation [63, 64], suggesting that some pDC activation-dependent changes in gene expression may be under circadian control of global TF expression. Along this line, Silver et al. showed that TLR9 function is controlled by the circadian molecular clock in a number of cell types including DCs [65]. Another group of TFs that show significant changes in expression after pDC activation could be classified as SMAD proteins, classical effectors of TGFβ signaling. It is known that stimulating DC progenitors with TGFβ accelerates DC differentiation, directing development toward cDCs [56]. Also, one of the SMAD proteins, SMAD3, has been determined as a key player in determining cDC versus pDC cell fates [57]. Interaction of SMAD proteins with known pDC driving factors such as Zeb2 have also been described [66, 67]. Other SMAD members do not affect pDC numbers, as shown in vivo in *Smad7*-deficient mice [68]. Further, TFs involved in various processes of the endoplasmic reticulum are differentially expressed in TLR9 activated pDCs. Notably, mouse and human pDCs are morphologically characterized by an extensive rough ER, enabling them to rapidly secrete copious amounts of type I IFN after TLR7 and TLR9 stimulation [69, 70]. The enrichment of TFs involved in mRNA binding processes, sumoylation and epigenetic modifications further highlights the changing biology of pDCs in protein production, posttranslational protein modifications, and alteration of the physical DNA structure that regulates gene expression after cell activation. We hereby define a novel set of expressed TFs in TLR9 activated pDCs, thus identifying TFs involved in particular biological processes that may require further investigation for their functional role in activated pDCs. The global transcriptomics approach allows a comparison for the expression patterns of several TFs belonging to the same TF family or involved in the same biological process, which may help to further narrow down interesting candidates.

Using CpG as an optimal TLR9 agonist and focusing on early events after virus infection, we found that after pDC activation more of the pDC chromatin landscape is "turned on" rather than "turned off“, both globally in the genome and also among the regions associated with TF genes themselves. Specifically, about 80% of all regions that show significant chromatin changes exhibited increased accessibility for TFs. However, with regard to gene expression, 2 h after pDC activation more genes were down-regulated than up-regulated as compared to the naïve state. One explanation could be that while DNA is more accessible, the TFs that possibly bind to these DNA stretches may inhibit rather than activate gene expression. Leylek et al. recently performed a global analysis of chromatin modulations in primary human DC populations including pDCs stimulated for 48 h by the TLR7 ligand imiquimod or CD40L [42]. In contrast to this study, we chose early time points after TLR9 activation with CpG (2 h, 6 h, 12 h), which represent immediate events after virus infection during which pDCs rapidly produce type I IFN to fight infection. Human pDCs stimulated with imiquimod for 48 h showed reduced protein and RNA expression of *TCF4* and *ZBTB18*, the latter was identified as a novel pDC specific TF in this paper [42]. We, however, observed significantly increased expression of *Tcf4* (2 h and 6 h vs steady state, respectively) and *Zbtb18* (2 h, 6 h and 12 h vs steady state, respectively). Our ATAC-Seq data show 11 regions with significantly more accessible chromatin within the *Tcf4* gene after 2 h CpG stimulation of pDCs, which may well explain the increased expression of the gene after TLR9 activation. *Zbtb18*, on the other hand, showed only one region downstream of the gene (+ 12,409) that is characterized by increased chromatin accessibility, but no significant chromatin changes in the promoter region or within the gene. The detected region downstream of *Zbtb18* may represent an enhancer region that regulates its gene expression but requires further verification. Leylek et al. reported ZBTB18 as a zinc finger TF to be specifically expressed and active in human pDCs [42]. Thus, the authors suggest it may regulate pDC gene expression as a so far little characterized factor for human pDC biology. Our data suggest in accordance with this study that ZBTB18 may also play a so far unrecognized but important role in the biology of mouse pDCs. An extensive motif analysis revealed that TFs belonging to the JAK-STAT and the NFκB signaling pathways exhibit increased accessibility to DNA binding regions after pDC stimulation. This underlines the importance of the JAK-STAT and NFκB signaling pathways in activated pDCs.

In contrast, Ets family members belonging to the Helix-turn-helix family of TFs and Zinc-coordinating zf-C2H2 TFs were both found to have less access to DNA after pDC activation. Ets family members include SPI1, also known as PU.1, which has been shown to drive the development of precursor cells toward cDC rather than pDC development [71]. Regarding pDC-driving cell fate TFs, IRF8 and RUNX2 belonging to the helix-turn-helix and β-scaffold TF groups, respectively, show motif enrichment in two sets of regions exhibiting increased versus decreased chromatin accessibility after pDC activation. Hence, cell fate TFs that drive pDC development both gain and lose access to distinct DNA regions after TLR9 activation.

Gene expression of the key pDC cell fate TFs IRF8, E2–2, and RUNX2 has been shown to steadily increase in expression during pDC precursor development into fully differentiated pDCs [34–38]. However, the role of these TFs for pDC survival and differentiation has not been investigated in detail after TLR9 activation. Here we observed different gene expression patterns for E2–2, and RUNX2 after pDC activation. E2–2 expression is strongly up-regulated at 2 h and 6 h of CpG stimulation vs no stimulation, but not at 12 h after CpG activation vs steady state. Runx2, on the other hand, is strongly down-regulated at each CpG stimulation time point as compared to the naïve state.

Our results therefore warrant further investigations of pDC cell fate TFs to explore the biological relevance of distinct expression patterns as well as the simultaneous gain and loss of accessibility to DNA by modulation of chromatin after pDC activation. We found that IRF7, NFκB1, and RELA as well as ATF3 and JUN, two AP-1 family members, fulfil three criteria relevant in this context: They exhibit (i) increased gene expression, (ii) enhanced chromatin accessibility for their gene regions, and (iii) enriched TF DNA binding motifs in the accessible genomic regions after pDC stimulation. We used this integrative omics approach to identify potential novel players important in pDC biology after cell activation. Increased gene expression (criterium i) of NFκB and IRF genes in TLR9 activated pDCs has been described already. It has been shown that pDCs sense MCMV via TLR9 mediated pathways [72] and that MCMV infection of mice resulted in increased NFkB, Irf1, Irf3, Irf7 and Irf8 expression in pDCs of infected mice as compared to uninfected mice [54]. According to our knowledge the impact of TLR9 activation on chromatin changes (criterium ii) within or upstream of NFκB and IRF genes has not been shown so far. An enrichment of NFκB motifs (criterium iii), however, has been described in B cells after cell activation [73]. Also while the biological role for IRF7, NFκB1, and RELA have been described in activated pDCs, there is little known about any function of AP-1 factors in pDCs which also fulfil all three criteria i-iii of our analysis. Activator Protein-1 (AP-1) was one of the first TFs to be described in the 1980s [74]. It consists of a dimeric protein complex with members from the JUN, FOS, ATF, BATF, or MAF protein families [75, 76]. A shared feature between the members is a basic leucine-zipper (bZIP) domain which is required for dimerization and DNA binding. The AP-1 family of TFs are known to regulate various biological processes such as proliferation, differentiation, and cell survival [76–79]. They have further been implicated in a variety of pathologies ranging from cardiovascular disease to cancer, hepatitis, and Parkinson’s disease [80–82]*.* A connection has been established between NFκB and AP-1 activity, which may be regulated by NFκB [83] suggesting a possible common molecular mechanism of these TFs in activated pDCs. Leylek et al. observed an increased TF activity score of AP-1 factors such as JUN and FOS after CD40L stimulation in human pDCs, which does not induce type I IFN expression [42]. They speculate that AP-1 factors may be necessary for the conversion of pDCs into cDC-like cells. Further, they suggest that AP-1 activity in primary human pDCs is repressed by TCF4 directly or indirectly. In our study, we observed increased expression and activity of AP-1 factors in mouse pDCs after TLR9 activation, which induces a robust type I IFN production. This suggests that AP-1 factors may play an important role in both human and mouse pDCs in the context of different type I IFN inducing or non-inducing stimuli. While Leylek et al. established a possible connection of AP-1 factors with DC differentiation, we suggest in addition a possible role of AP-1 factors in type I IFN production in pDCs. In fact, AP-1 has already been shown to be required for spontaneous type I IFN production in pDCs. whereas type I IFN production triggered by pathogen receptor recognition such as TLR stimulation was not affected by AP-1 inhibition [84]. In contrast, our in silico analyses suggest a close link between AP-1 factors and pDC biology after TLR9 stimulation: The AP-1 motif is present within the open chromatin region of the proximal promoter site of the *Tcf4* gene, a prominent pDC cell fate TF. Grajkowska et al. showed that there are two *Tcf4* isoforms, the expression of which is controlled during pDC differentiation by two respective promoters as well as distal enhancer regions within 600–900 kb 5′ and ~ 150 kb 3′ of the *Tcf4* gene [85]. However, the binding site of specific TFs to these cis-regulatory sites has not been fully evaluated. This calls for further investigations on the AP-1 binding site in activated pDCs newly identified in our study. One of the key AP-1 candidates in our investigation, ATF3, has been described as a negative regulator of antiviral signaling in Japanese encephalitis virus infection in mouse neuronal cells [86]. The hallmark of pDCs is their importance in antiviral immune responses, pointing toward ATF3 as an interesting candidate to investigate in TLR9 activated pDCs. Another AP-1 family member, JUN, was the first oncogene to be described [87] and has since been studied in detail in the context of various tumor entities. In contrast, knowledge about its role in the context of infection is limited. For example, it has been shown to have a regulatory role in H5N1 influenza virus replication and host inflammation in mice [88]. Our analyses revealed a distinct regulation of *Jun* expression and chromatin structure combined with an increased global DNA binding accessibility in pDCs after activation. Further studies are required to assess the role of *Jun* regulation in pDCs upon a microbial stimulus or in a chronically activated state that might unravel unknown functions of this TF in immunity. While targeting TFs for therapeutic purpose has been proven difficult so far, recent advances have been made through novel chemistries and the use of staples peptides to disrupt protein-protein interactions [89, 90]. Thus, the in silico analyses of the global TF reservoir in pDCs from our study led to the identification of novel candidates that warrant further investigation regarding their role in pDC biology, in particular after cell activation, which may lead to the development of novel therapeutics to treat infection, autoimmune disease and cancer.

## Conclusions

In the present study, we performed a detailed analysis on the changes in expression and chromatin accessibility for the complete set of all known TFs in pDCs for early time points after activation. To this purpose, we used the AnimalTFDB data base and combined RNA-Seq, ATAC-Seq, and Gene Ontology analyses to define global TF gene expression, chromatin landscapes, and biological pathways in these cells. Based on our findings, we suggest a newly defined set of TLR9-dependent TFs associated with pDC activation. We further identify the AP-1 family of TFs, which are so far less well characterized in pDC biology, as possibly important players in these cells after activation.

## Methods

### Mice

C57BL/6N mice were originally obtained from Charles River Laboratories and housed under specific pathogen-free conditions in the animal research facility of the University of Düsseldorf strictly according to the German Animal Welfare Act. The mice were euthanized by cervical dislocation before bone marrow was harvested. All experiments were performed using bone marrow from sex and age matched littermates between 7 to 14 weeks of age.

### Generation and stimulation of BM-derived pDCs for RNA-Seq and ATAC-Seq

BM-derived Flt3-L cultured pDCs were generated as previously described [91]. For RNA-Seq, BM-derived pDCs (CD3^−^CD19^−^CD11c^+^CD11b^low^B220^+^SiglecH^+^ CD317^+^) were FACS purified using FACS Aria III (BD). The pDCs were left untreated or stimulated with 1 μM CpG 2216 (Tib Molbiol, Nr. 930,507 l) complexed to transfection reagent DOTAP (Roche) for 2 h, 6 h or 12 h. RNA was isolated by using the NucleoSpin II RNA mini kit (Macherey-Nagel) and subjected to RNA-Seq. For ATAC-Seq BM-derived pDCs (CD3^−^CD19^−^CD11c^+^CD11b^low^B220^+^SiglecH^+^CD317^+^) were FACS purified using FACS Aria III (BD). The pDCs were left untreated or stimulated with 1 μM CpG 2216 complexed to transfection reagent DOTAP (Roche) for 2 h. At the end of stimulation time, cells were kept on ice and stained for 7AAD (BD). Live cells (7AAD^−^) were further purified by FACS and kept frozen in complete RPMI medium containing 5% DMSO. The frozen cells were transported on dry ice to Active Motif (Belgium) for ATAC-Seq.

The following antibodies have been used: CD3-PerCP (BD Bioscience, Clone: 145-2C11), CD19-PerCP-Cy5.5 (BD Bioscience, Clone:1D3), CD11c-PE-Cy7 (BioLegend, Clone: N418), CD11b-APC-Cy7 (BD Bioscience, Clone: M1/70), B220-FITC (BD Bioscience, Clone: RA3-6B2), SiglecH-APC (BioLegend, Clone 551), CD317-PE (eBioscience/Thermoscientific, Clone: ebio927).

### RNA-Seq analyses

DNase digested total RNA samples used for transcriptome analyses were quantified (Qubit RNA HS Assay, Thermo Fisher Scientific) and quality measured by capillary electrophoresis using the Fragment Analyzer and the ‘Total RNA Standard Sensitivity Assay’ (Agilent Technologies, Inc. Santa Clara, USA). All samples in this study showed high RNA Quality Numbers (RQN; mean = 9.9). The library preparation was performed according to the manufacturer’s protocol using the Illumina® ‘TruSeq Stranded mRNA Library Prep Kit’. Briefly, 200 ng total RNA were used for mRNA capturing, fragmentation, the synthesis of cDNA, adapter ligation and library amplification. Bead purified libraries were normalized and sequenced on the HiSeq 3000/4000 system (Illumina Inc. San Diego, USA) with a read setup of SR 1 × 150 bp. The bcl2fastq tool was used to convert the bcl files to fastq files as well for adapter trimming and demultiplexing.

Data analyses on fastq files were conducted with CLC Genomics Workbench (version 11.0.1, QIAGEN, Venlo. NL). The reads of all probes were adapter trimmed (Illumina TruSeq) and quality trimmed (using the default parameters: bases below Q13 were trimmed from the end of the reads, ambiguous nucleotides maximal 2). Mapping was done against the *Mus musculus* (mm10; GRCm38.86) (March 24, 2017) genome sequence. Samples (three biological replicates each) were grouped according to their respective experimental condition. Raw counts were next re-uploaded to the Galaxy web platform. The public server at usegalaxy.org was used to perform multi-group comparisons [92]. Differential expression of genes between any two conditions was calculated using the edgeR quasi-likelihood pipeline which uses negative binomial generalized linear models with F-test [93, 94]. Low expressing genes were filtered with a count-per-million (CPM) value cut-off that was calculated based on the average library size of our RNA-Seq experiment [43]. The resulting *p* values were corrected for multiple testing by the false discovery rate (FDR) [95]. A p value of < 0.05 was considered significant. RNA-Seq data are deposited with NCBI’s Gene Expression Omnibus (GEO) and are accessible through GEO Series accession number GSE170750 ( https://www.ncbi.nlm.nih.gov/geo/query/acc.cgi?acc=GSE170750 ).

### ATAC-Seq

Cells were harvested and frozen in culture media containing FBS and 5% DMSO. Cryopreserved cells were sent to Active Motif to perform the ATAC-Seq assay. The cells were then thawed in a 37 °C water bath, pelleted, washed with cold PBS, and tagmented as previously described [96], with some modifications [97]. Briefly, cell pellets were resuspended in lysis buffer, pelleted, and tagmented using the enzyme and buffer provided in the Nextera Library Prep Kit (Illumina). Tagmented DNA was then purified using the MinElute PCR purification kit (Qiagen), amplified with 10 cycles of PCR, and purified using Agencourt AMPure SPRI beads (Beckman Coulter). Resulting material was quantified using the KAPA Library Quantification Kit for Illumina platforms (KAPA Biosystems), and sequenced with PE42 sequencing on the NextSeq 500 sequencer (Illumina).

Reads were aligned using the BWA algorithm (mem mode; default settings). Duplicate reads were removed, only reads mapping as matched pairs and only uniquely mapped reads (mapping quality ≥1) were used for further analysis. Alignments were extended in silico at their 3′-ends to a length of 200 bp and assigned to 32-nt bins along the genome. The resulting histograms (genomic “signal maps”) were stored in bigWig files. Peaks were identified using the MACS 2.1.0 algorithm at a cut off of *p*-value 1e-7, without control file, and with the –nomodel option. Peaks that were on the ENCODE blacklist of known false ATAC-Seq peaks were removed. Signal maps and peak locations were used as input data to Active Motifs proprietary analysis program, which creates Excel tables containing detailed information on sample comparison, peak metrics, peak locations, and gene annotations. For differential analysis, reads were counted in all merged peak regions (using Subread), and the replicates for each condition were compared using DESeq2. ATAC-Seq data are deposited with NCBI’s GEO and are accessible through GEO Series accession number GSE171075 (https://www.ncbi.nlm.nih.gov/geo/query/acc.cgi?acc=GSE171075).

### Downstream analyses and visualization of omics data

Volcano plots were created using ggplot2 [98] and ggrepel [99]. Heatmaps were created using Morpheus (https://software.broadinstitute.org/morpheus). Pearson correlation matrices were calculated in R and plotted as heatmaps using gplots [100]. Pathway analyses for different gene ontology (GO) terms and subsequent functional classification and annotation clustering were performed using the Database for Annotation, Visualization and Integrated Discovery (DAVID) [101]. Venn diagrams were created using R package eulerr [102]. Evolutionary conserved regions (ECR) for selected genes were shown by taking a screenshot from the ECR browser [103]. Bar graphs were plotted in Gradphpad Prism version 8.4.3 on Windows (GraphPad Software, La Jolla California USA, www.graphpad.com). ATAC-Seq peaks were visualized using IGV [104, 105].

### TF motif analyses

ATAC-Seq regions that indicated differentially accessible chromatin regions between naive and 2 h CpG stimulated samples (DESeq2, |FC| > 2, *p* < 0.05) were used for motif analysis. The regions were adjusted to the same size (500 bp). The MEME-Centrimo differential motif analysis pipeline [106] was run on the fasta files representing each chromatin region (significantly increased vs decreased chromatin access after CpG stimulation) to identify overrepresented motifs, using default parameters and the HOCOMOCO v11 motif database. The search for the occurrences of the AP-1 motif, and motifs of early-responding genes as well as signaling dependent TF motifs among selected DNA sequences was performed with MEME-FIMO.

## Supplementary Information

**Additional file 1: Supplemental Table S1.** FIMO analysis of immediate signaling-dependent TF motifs among promoters of early-responding TFs that are more expressed at 2 h vs steady state.

**Additional file 2: Supplemental Table S2.** FIMO analysis of immediate signaling-dependent TF motifs among promoters of early-responding TFs that are less expressed at 2 h vs steady state.

**Additional file 3: Supplemental Table S3.** FIMO analysis of early-responding TF motif presence in promoters of up-regulated late-responding TF genes.

**Additional file 4: Supplemental Table S4.** FIMO analysis of early-responding TF motif presence in promoters of down-regulated late-responding TF genes.

**Additional file 5: Supplemental Table S5.** Functional cluster analysis with 661 CpG-dependent TF genes.

**Additional file 6: Supplemental Table S6.** Centrimo motif enrichment analysis using the HOCOMOCO database and 2174 gene promoter associated regions with increased chromatin accessibility after pDC activation.

**Additional file 7: Supplemental Table S7.** FIMO analysis of immediate signaling-dependent TF motifs among promoters of early-responding TFs that are more expressed and have more accessible chromatin at 2 h vs steady state.

## Data Availability

RNA-Seq and ATAC-Seq data sets analysed during the current study are available in the NCBI’s Gene Expression Omnibus (GEO) depository and are accessible through GEO Series accession number GSE170750 (https://www.ncbi.nlm.nih.gov/geo/query/acc.cgi?acc=GSE170750). and GSE171075 (https://www.ncbi.nlm.nih.gov/geo/query/acc.cgi?acc=GSE171075), respectively.

## Abbreviations

bZIP: Basic leucine-zipper
BP: Biological processes
BM: Bone marrow
casc: Cascade
CC: Cellular components
CPM: Count-per-million
Cyt: Cytokine
Db: Databases
DAVID: Database for Annotation, Visualization and Integrated Discovery
DC: Dendritic cell
DETFs: Differentially expressed TFs
FDR: False discovery rate
GEO: Gene Expression Omnibus
GO: Gene Ontology
Horm: Hormone
IRF8: Interferon regulatory factor 8
Med: Mediated
MF: Molecular functions
IPCS: Natural interferon (IFN)-producing cells
Otx: Orthodenticle homeobox
pDCs: Plasmacytoid dendritic cells
Reg: regulation
rERs: Response to endoplasmic reticulum stress
Resp: Response
SRF: Serum response factor
Sig: Signaling
STAT: Signal Transducers and Activators of Transcription
SF: Steroidgenic factor
TFs: Transcription factors
TLRs: Toll like receptors
TSC22: Transforming growth factor-β stimulated clone-22
TSS: Transcriptional start site
